# Optimize the Preparation of Novel Pyrite Tailings Based Non-sintered Ceramsite by Plackett-Burman Design Combined With Response Surface Method for Phosphorus Removal

**DOI:** 10.3389/fchem.2022.850171

**Published:** 2022-03-08

**Authors:** Ruihuan Chen, Zhenlin Pan, Shuyi Chu, Jibo Xiao, Rengui Weng, Da Ouyang, Yunlong Yang, Xiangting Wu, Zhida Huang

**Affiliations:** ^1^ College of Life and Environmental Science, Wenzhou University, Wenzhou, China; ^2^ Zhejiang Provincial Key Laboratory for Water Environment and Marine Biological Resources Protection, Wenzhou University, Wenzhou, China; ^3^ Wenzhou Academy of Agricultural Sciences, Wenzhou, China; ^4^ Indoor Environment Engineering Research Center of Fujian Province, Fujian University of Technology, Fuzhou, China; ^5^ Key Laboratory of Soil Contamination Bioremediation of Zhejiang Province, School of Environmental and Resource Sciences, Zhejiang Agriculture and Forestry University, Hangzhou, China; ^6^ Wenzhou Institute of Industry and Science, Wenzhou, China

**Keywords:** pyrite, solid waste recycling, unburned ceramsite, phosphorus removal, filter material, harmless treatment

## Abstract

The large amount of untreated pyrite tailings has caused serious environmental problems, and the recycling of pyrite tailings is considered as an attractive strategy. Here, we reported a novel non-sintered ceramsite prepared with pyrite tailings (PTNC) as the main active raw material for phosphorus control, and the dosage effect of ingredients on total phosphorus (TP) removal ability was investigated. The results from Plackett-Burman Design (PBD) suggested the dosages of dehydrated sludge, sodium bicarbonate, and cement were the factors which significantly affect the TP removal ability. The Box-Behnken Design (BBD) based response surface methodology was further employed, and it indicated the interactions between different factors, and the optimized recipe for PTNC was 84.5 g (pyrite tailings), 10 g (cement), 1 g (calcined lime), 1 g (anhydrous gypsum), 3 g (dehydrated sludge), and 0.5 g (sodium bicarbonate). The optimized PTNC was characterized and which presented much higher specific area (7.21 m^2^/g) than the standard limitation (0.5 m^2^/g), as well as a lower wear rate (2.08%) rather than 6%. Additionally, the leaching metal concentrations of PTNC were far below the limitation of Chinese National Standard. The adsorption behavior of TP on PTNC was subsequently investigated with batch and dynamic experiments. It was found that the calculated max adsorption amount (q_max_) was about 7 mg/g, and PTNC was able to offer a stable TP removal ability under different hydraulic retention time (HRT). The adsorption mechanism was discussed by model fitting analysis combined with XRD and SEM characterization, and cobalt phosphide sulfide was observed as the newly formed substance through the adsorption process, which suggested the existing of both physical and chemical adsorption effect. Our research not only offered an economic preparation method of ceramsite, but also broadened the recycling pathway of pyrite tailings.

## 1 Introduction

Pyrite is the most abundant and widespread sulfide mineral which is mainly composed of iron and sulfur, and has been used as the raw material for sulfuric acid production for decades (Chandra and Gerson 2010). However, with the large-scale mining of pyrite, the pyrite tailings have caused serious environmental problems. As sulfide-rich waste materials, pyrite tailings can be oxidized in the presence of air and water (Garcia et al., 2005) spontaneously, which causes acid mine drainage (AMD) consequently (Malmström et al., 2006). AMD is considered as a serious and persistent environmental problem, which leads to the environment acidification and also releasing of significant amounts of various toxic metals into surface and groundwater (Heikkinen and Räisänen, 2009; Wang and Mulligan, 2009; Sahoo et al., 2013; Carvalho et al., 2014). Thus, the control of the pyrite tailings sourced pollution has become an important issue in the past decades, and various methods have been reported (Ouyang et al., 2015; Park et al., 2019; Dong et al., 2020a).

Remediation strategies are effective but unsustainable; thus, recycling of mine tailings is considered as a more economical way for reducing of tailings amount and limiting acid mine drainage formation. Currently, the most popular strategy for tailings recycling is replacing some portion of traditional but expensive construction materials, such as concrete admixture (Guo et al., 2016) and road construction admixture (Oluwasola et al., 2015). However, drawbacks still hindered the application of these strategies, such as higher energy consumption, dust generation increasing, and CO_2_ emission (Khale and Chaudhary, 2007), and novel effective and environment friendly method towards pyrite tailings recycling should be proposed. One important property of pyrite tailings should not be ignored, that is, large amounts of active reaction sites due to the existing of Fe and S in pyrite tailings, which can be used for removal of various pollutants (Yang et al., 2017). Thus, it is promising to use pyrite tailings as the active material to enhance the wastewater treatment process.

It is well known that the effluents of municipal wastewater treatment plants (WWTPs) contributed highly to these elements’ concentrations in surface water, and the abundant nutrition elements (especially phosphorus) in water have caused a serious eutrophication problem in the past decades (Schindler et al., 2016). Thus the resource utilization and advanced treatment of waste water is a very meaningful way for the sustainable development of human beings, and strategies have been proposed (Bashar et al., 2018; Zeng et al., 2021). The biological aerated filter system (BAFS) has gained increasing attention due to smaller footprint and favorable ability for contaminants removal, which presented effective removal effect on COD, NH_3_-N, and TN (Dong et al., 2020b). BAFS generally takes advantages of a granular media for the formation of microbial biofilms and provides the depth filtration action as well. Certainly, the biomass, bioactivity, and filtration action in BAFS is highly depending on the media character, and it also significantly determines the investment of construction and operating cost. Thus, research has reported focusing on the investigation of media performance of different materials in BAFS (Qiu et al., 2010; Bao et al., 2016; Bao et al., 2019), and ceramic particle was found as a favorable candidate due to its large specific surface area, high mechanical strength, and biological affinity (Yue et al., 2009; Han et al., 2013; Yang et al., 2015). However, the phosphorus removal by commercial ceramsites was unfavorable as yet (Jiang et al., 2014), and it is significant to propose strategies to improve its phosphorus removal.

Ceramsite was generally prepared with various active materials such as clay (Zhang et al., 2019), activated sludge (Nie et al., 2021) and fly ash (Mi et al., 2021) by high temperature calcination, which not only aim to improve the material properties and safety, but also promote the recovery and utilization of waste resource. However, the calcination is a high energy consumption process, and the crystal structure of raw material would be destroyed leading to the decrease of active adsorption sites. Consequently, non-sintered is considered as a more economic and effective way for preparation of ceramsites. Shao et al. (2019) prepared a non-sintered fly ash ceramsite for ammonia nitrogen adsorption, and which showed favorable adsorption capacity (4.25 mg/g) and standard-compliant leaching toxicity. Li et al. (2015) investigated the performance of non-sintered fly-ash ceramsite in the dual membrane processes for treatment of ethylene chemical plant wastewater, which indicated the biological aerated filter loaded with non-sintered ceramsite was a reasonable and effective method for pretreatment of reverse osmosis process. Besides fly-ash, the raw materials for non-sintered ceramsite preparation can be various, which provides more opportunities for recycling of solid wastes (He and Wang, 2019; Wang et al., 2021). Previous reports have already proved pyrite as a favorable absorbent candidate for wastewater treatment (Xu et al., 2006; Bulut et al., 2014; Ge et al., 2019; Chero-Osorio et al., 2021), and it is attractive to use pyrite tailings as the raw material for preparation of novel effective ceramsite towards phosphorus removal.

To our knowledge, there is no available report about the investigation of pyrite tailings based ceramsite as yet; thus, in this research, we employed pyrite tailings as the major active material aiming to prepare a novel non-sintered ceramsite (PTNC) for phosphorus control in wastewater treatment process. The main objectives are 1) investigate and optimize the recipe of raw materials mass ratio; 2) characterize the optimized PTNC with XRD, BET, SEM, and confirm its security by leaching toxicity metal concentration determination; and 3) investigate the application potential and clarify the removal mechanism of total phosphorus (TP) with batch adsorption experiment and dynamic column experiment.

## 2 Materials and Methods

### 2.1 Materials and Chemicals

Pyrite tailings were collected from Anyang, Henan Province, and the chemical composition and XRD data of pyrite tailings are shown in Supplementary Table S1 and Supplementary Figure S1 respectively. The commercial 425^#^ cement was used as adhesive and stabilizer, and calcium lime (≥60% effective calcium oxide) and anhydrous gypsum (industrial grade) were used as activator. Dehydrated sludge (Supplementary Table S2) is obtained from a sewage treatment plant in Wenzhou and used to reduce the product weight. Sodium bicarbonate (analytical grade) was selected as the pore-forming agent.

### 2.2 Preparation of PTNC

Firstly, 25 ml deionized water was added into a mixture (totally 100 g) containing pyrite tailings, cement, calcium lime, anhydrous gypsum, sodium bicarbonate, and dehydrated sludge with required mass ratio. After pelleting to spherical ceramsite (6–10 mm), it was air-dried for 12 h at room temperature, then the ceramsites were steam cured with an autoclave at 1.2 MPa and 100°C for 10 h. The final PTUC product was obtained by regularly curing with spraying water for 2 days.

### 2.3 Optimization of the Components Dosage

#### 2.3.1 Single Factor Experiment

Single factor experiments were performed to preliminary define the dosage of raw materials, and the phosphate removal rate as well as porosity were selected as the indicators. For each single experiment, the total mixture weight was fixed as 100 g, and the amount of pyrite tailings varied with the changing of corresponding ingredient amount. The test amounts of each material were shown as follows: cement (10, 20, 30, 40, and 50 g), calcium lime (1, 2, 3, 4, and 5 g), anhydrous gypsum (1, 2, 3, 4, and 5 g), dehydrated sludge (2, 4, 6, 8, and 10 g), and sodium bicarbonate (05, 1, 2, 3, 4, and 5 g).

#### 2.3.2 Plackett-Burman Experiments

In order to obtain the significant influence factors on the phosphate removal capacity and porosity of PTNC, PBD method was used and the design scheme can be found in Supplementary Table S3, where five ingredients were coded respectively from code A to E, phosphate removal capacity was set as Y.

#### 2.3.3 Box-Behnken Design Based Response Surface Methodology

Based on the significantly influence factors obtained from PBD experiments, the dosage of dehydrated sludge, sodium bicarbonate, and cement were selected and coded as X_1_, X_2_, and X_3_ respectively for Box-Behnken design to optimize the PTNC preparation process. Phosphate removal capacity (Y) was used as the indicators, and the design scheme is shown in Supplementary Table S4.

### 2.4 Batch Adsorption Experiment for TP Removal

A total of 4.5 g PTUC was weighted and added into the conical flask (150 ml) and 50.0 ml solution containing required concentration of potassium dihydrogen phosphate was added, and the vials were shaken at 100 rpm for 5 h at 25°C. The TP concentration in the filtered liquid was determined by Mo-Sb anti-spectrophotometry method (λ = 700 nm). The effect of pH, PTNC dosage, co-existing anions, and temperature on adsorption were investigated respectively as well as the adsorption kinetic, and all the experiments were performed in triplicate vials. The removal rate of TP was calculated as follow equation:

$$\mathrm{TP}\;\mathrm{removal}\;\mathrm{rate}=\frac{c_{1}-c_{0}}{c_{0}}\;\times 100\text{\% }$$ (1)

where c_0_ and c_1_were the initial TP and filtered TP concentration in aqueous respectively.

### 2.5 Dynamic Adsorption of Total Phosphorus by PTNC in Column Experiment

In order to evaluate to the application potential of PTNC in TP control, an organic glass column (50 × 500 mm) was used to perform the dynamic adsorption experiment. PTNC was prepared according to the optimized results and filled into the column to 400 mm height. Simulated wastewater containing 26 mg/L TP was pumped into the column from the column bottom by a peristaltic pump, and the effect of hydraulic retention time (HRT) on TP removal was investigated in the range of 1–3 h. The diagram of experimental device can be found in Supplementary Figure S2.

### 2.6 Characterization of PTNC

The physicochemical properties such as bulk density, apparent density, crush/wear rate, solubility in hydrochloric acid, voidage, and silt content were tested according to the Chinese standard (CJ/T 299-2008). Water absorption and cylinder compressive strength were tested as Chinese national standard (GB/T 17,431.2-2010). X-ray diffraction (XRD), BET method, and scanning electron microscopy (SEM) were employed to analyze the changes of crystal structure, specific surface area, and morphology after adsorption.

The leaching analysis of heavy metal was performed to evaluate the potential risk of PTNC. Briefly, sulfuric acid and nitric acid were mixed (mass ratio 2:1), and the added to deionized water and the pH was adjusted at 3.2. Then, 100 g PTNC was mixed with 1 L liquid above and shaken at 25°C, 30 rpm, for 20 h. After filtered with 0.45 μm membrane, the concentration of heavy metals was determined by ICP-OES, and the results were compared with the limitation of the standard of Leaching Toxicity Identification of Hazardous Waste (GB5085.3-2007).

## 3 Results and Discussion

### 3.1 Single Factor Experiments

**FIGURE 1:**
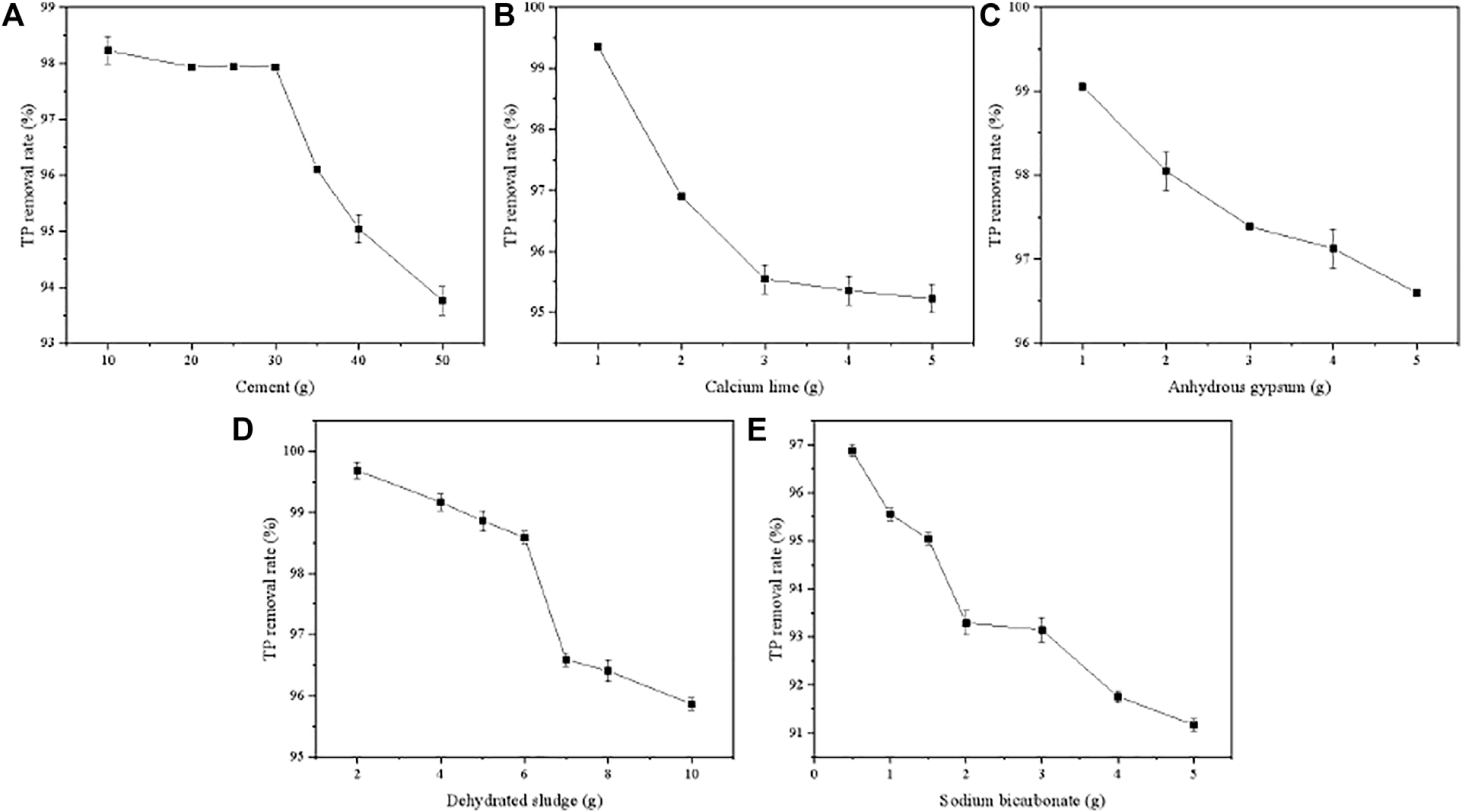
Effect of ingredients on TP removal rate of PTNC.

Figure 1 showed the effect of five ingredients on the TP removal capacity of PTNC. In terms of cement, the TP removal rate decreased from 98.23 to 95.04% with the increasing of cement content from 10 to 50 g (Figure 1A). It can be found that the removal rate was stable in the dosage range of 10–30 g, which can attributed to the relative high amount of Ca (over 60%) that react with PO_4_^3-^ to form a complex compound. As the dosage of cement was over 30 g, the decreasing of removal rate can be ascribed to the lower porosity of PTNC, which was almost 0 as 50 g cement was added. The TP removal rate decreased from 99.35 to 95.23% with the increasing of calcium lime (Figure 1B). Adding of quicklime can increase the content of Ca in ceramsite to some extent, which is beneficial to the removal of TP. However, the excess using of quicklime will destroy the Si-O bond in the product due to the alkaline environment, which leads to the reduction of the specific surface area. Therefore, the dosage of quicklime was selected as 1 g. With the increasing of gypsum dosage from 1 to 5 g, the TP removal rate decreased from 99.05 to 96.60% (Figure 1C). The main component of gypsum is CaSO_4_ which could increase the content of Ca in ceramsite, and was conducive to the removal of TP, but the excessive amount of gypsum leads to a longer digestion time of quicklime due to its high hygroscopicity, which reduces its activity and leads to a decrease in specific surface area and porosity. Therefore, the amount limitation of PTUC gypsum is set as 1 g. In the terms of dehydrated sludge (Figure 1D), the TP removal rate showed decreasing tendency as the dosage increased. It can be ascribed to the high amount of organic matter that inhibited the hydration reaction and hindered the production of gelled substances, which resulted in a decrease in porosity (Figure 1E). A similar tendency can be found in the effect of sodium bicarbonate. Adding sodium bicarbonate can promote the forming of pores structures during steam curing, but too much sodium bicarbonate will release a large amount of CO_2_ at high temperature, which makes the skeleton structure become loose and difficult to form into pellets. Thus, the sodium bicarbonate dosage is set as 0.5 g here.

### 3.2 Significant Factors Affecting the TP Removal Rate

**FIGURE 2:**
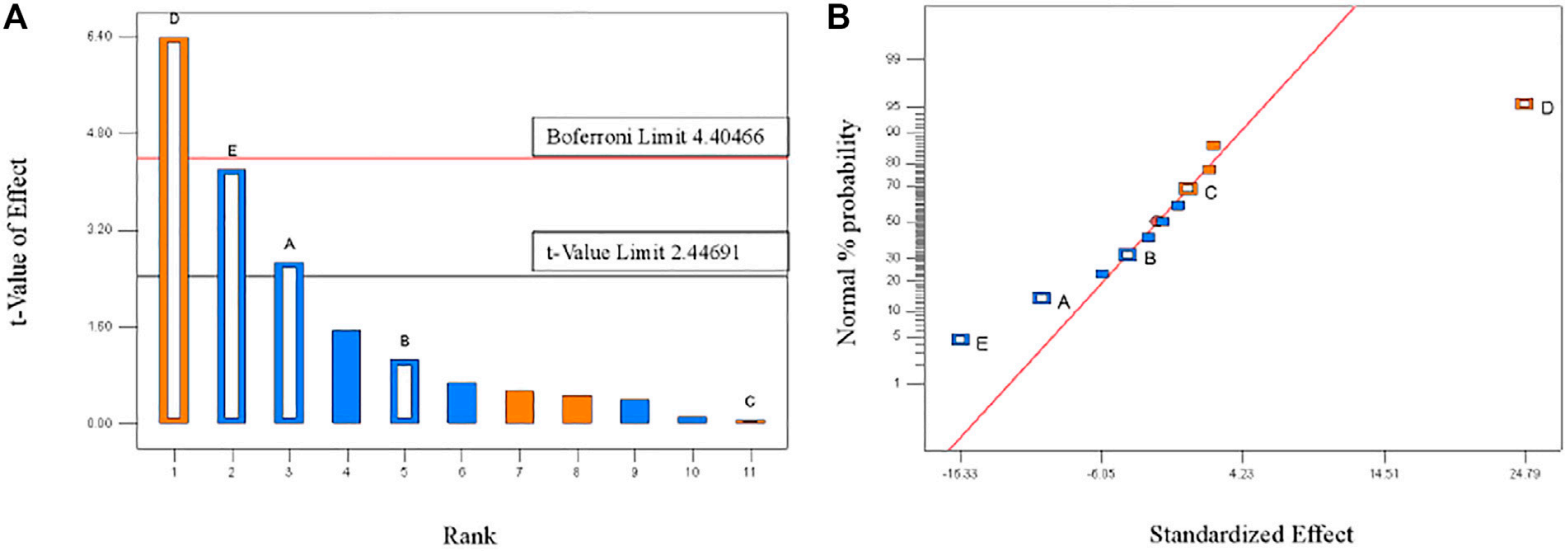
Pareto plot **(A)** and normal distribution plot **(B)** of TP removal rates. The hollow square column represents the experimental data, and the solid square column represents the dummy error. The red line in Pareto plot indicates the 99% confidence interval limitation and black line stands for 95% confidence interval limitation.

Based on the results of a signal factor experiment, a two-level PBD factorial design of 12 runs was employed to unbiasedly screen the variables that significantly affect the TP removal rate by PTNC (Table 1), and Figure 2 showed the analysis results. Five ingredients affected the TP removal rate as the following order: D (Dehydrated sludge) >E (Sodium bicarbonate) >A (Cement) >B (Calcium lime) >C (Anhydrous gypsum), and the dosage of dehydrated sludge affected TP removal rate most significantly which arrived at the 99% confidence interval. Sodium bicarbonate and cement also showed significant influence effect on TP removal rate, which were observed at 95% confidence interval (Figure 2A). It can be also proved by the normal distribution plot (Figure 2B), where the points standing for dehydrated sludge, sodium bicarbonate, and cement showed significant dispersion from the fitted line. Table 2 shows the results of significant inspection of regression, which further proved the factors with significant effect on the TP removal rate were dehydrated sludge, sodium bicarbonate, and cement, and a model Equation 1 for TP removal rate (%) (Y) was proposed; the *p* value and *R* ^2^ for the model were 0.0366 and 0.9179 which indicated the validity of the model.

Y=68.91−5.19A−2.06B+0.14C+12.40D−8.17E (2)

**TABLE 1:** Experimental design and response of Plackett-Burman experiment.

Runs	A	B	C	D	E	Removal rate (%)
1	+1	+1	−1	+1	+1	56.77
2	−1	+1	+1	−1	+1	51.37
3	+1	−1	+1	+1	−1	92.84
4	−1	+1	−1	+1	+1	85.68
5	−1	−1	+1	−1	+1	52.83
6	−1	−1	−1	+1	−1	92.14
7	+1	−1	−1	−1	+1	49.71
8	+1	+1	−1	−1	−1	58.05
9	+1	+1	+1	−1	−1	56.86
10	−1	+1	+1	+1	−1	92.30
11	+1	−1	+1	+1	+1	68.05
12	−1	−1	−1	−1	−1	70.23

**TABLE 2:** Significant test for Plackett-Burman design regression model.

	Factors	Adj SS	DF	Adj MS	F	p
TP removal rate	Model	3,018.45	5	603.69	13.41	0.0033
	A	323.00	1	323.00	7.18	0.0366
	B	51.07	1	51.07	1.13	0.3278
	C	0.23	1	0.23	0.00518	0.9451
	D	1843.70	1	1843.70	40.96	0.0007
	E	800	1	800.44	17.78	0.0056
	Error	270.05	6	45.01	—	—
	Total	3,288.50	11	—	—	—

Adj SS, Adjusted Sum of Square; Adj MS, Adjusted Mean Square; DF, Degrees of Freedom, (Wang et al., 2014).

### 3.3 Box-Behnken Design for Preparation Optimization

Based on the PBD results, the significant factors dehydrated sludge (X_1_), sodium bicarbonate (X_2_), and cement (X_3_) were considered for further optimization using BBD, and the matrix for BBD along with the experimental results is shown in Table 3. By applying multiple regression analysis on the experimental data, the following second order polynomial model was obtained to describe the TP removal rate (2):

$$\text{Y}=+64.90+14.71{\text{X}}_{1}-3.48{\text{X}}_{2}-5.55{\text{X}}_{3}-1.99{\text{X}}_{1}{\text{X}}_{2}+3.24{\text{X}}_{1}{\text{X}}_{3}+1.61{\text{X}}_{3}{\text{X}}_{2}-4.65{\text{X}}_{1}^{2}+12.48{\text{X}}_{2}^{2}+8.42{\text{X}}_{3}^{2}$$ (3)

**TABLE 3:** Experimental design and results of BBD.

Run order	X_1_	X_2_	X_3_	TP removal rate (%)
1	−1	−1	0	60.45
2	1	−1)	0	89.69
3	−1	+1	0	59.74
4	+1	+1	0	81.03
5	−1	0	−1	64.90
6	+1	0	−1	92.00
7	−1	0	+1	38.85
8	+1	0	+1	78.92
9	0	−1	−1	93.34
10	0	+1	−1	80.88
11	0	−1	+1	87.50
12	0	+1	+1	81.47
13	0	0	0	57.32
14	0	0	0	60.15
15	0	0	0	70.93
16	0	0	0	69.66
17	0	0	0	66.45

The adequacy of the model was checked using ANOVA and the results were shown in Table 4. The F value of model was 7.54 and the *p* value (Prob>F) was 0.018, indicating that the model was highly significant, which was also confirmed by the non-significant *p* value of “Lack of fit.” It can be also found that the variable X_1_ as well as the quadratic terms X_2_^2^ and X_3_^2^ showed significant relationship with the TP removal rates. *R* ^2^ of the model was 0.9065, suggesting the model has a good agreement for data fitting. The ratio of signal to noise (Adeq Precision) was 9.738 which was over 4, and the coefficient of variation (C.V.) was lower than 10%, indicating the reproducibility of the model. These statistical analysis results showed that the model was reliable and accurate, and can be used for the analysis and prediction of TP removal by PTNC. The statistical model was further validated by experiments with PTNC prepared under different conditions (Supplementary Figure S3). It can be said that the predicted model response for experimental value was close to the predicted value, thus validating the model.

**TABLE 4:** ANNOVA for BBD.

Source	Adj SS	DF	Adj MS	F-value	*p*-value
Model	3,197.96	9	355.32	7.54	0.0072
X_1_	1731.58	1	1731.58	36.75	0.0005
X_2_	97.01	1	97.01	2.06	0.1945
X_3_	246.29	1	246.29	5.23	0.0561
X_1_X_2_	15.80	1	15.80	0.34	0.5806
X_1_X_3_	42.03	1	42.03	0.89	0.3764
X_2_X_3_	10.31	1	10.31	0.22	0.6541
X_1_^2^	91.11	1	91.11	1.93	0.2070
X_2_^2^	655.62	1	655.62	13.91	0.0074
X_3_^2^	298.33	1	298.33	6.33	0.0400
Error	329.85	7	47.12	—	—
Lack of fit	188.36	3	62.78	1.78	0.2908
Pure error	141.48	4	35.37	—	—
Total	3,527.81	16	—	—	—

Adj SS, Adjusted Sum of Square; Adj MS, Adjusted Mean Square; DF, Degrees of Freedom, (Wang et al., 2014).

Coefficient of variation - 9.46%; Signal to noise ratio - 9.738; *R* ^2^ - 0.9065; R^2^_Adj_ - 0.7863.

**FIGURE 3:**
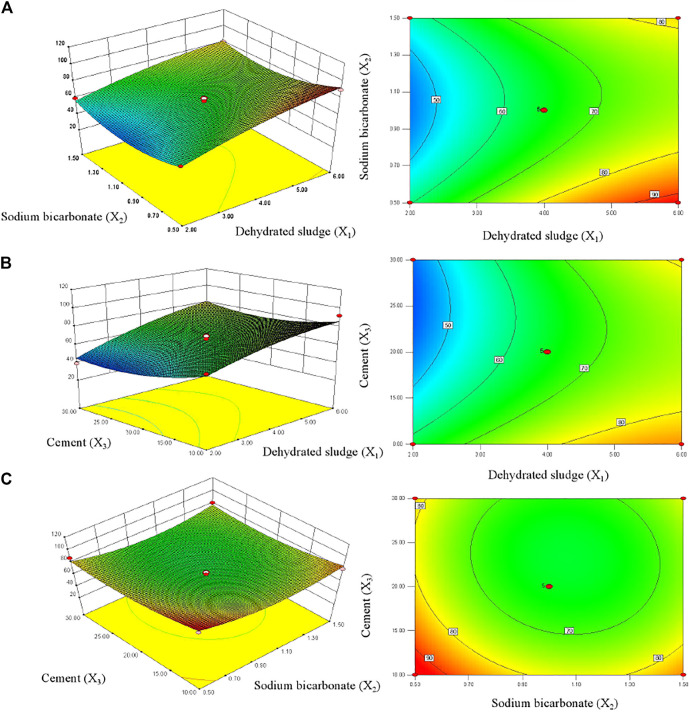
Surface and contour plot showing interactions between variables on TP removal rates by PTNC. **(A)** stands for interaction between dehydrated sludge and sodium bicarbonate; **(B)** shows interaction between dehydrated sludge and cement; **(C)** stands for interaction between sodium bicarbonate and cement.

The optimum level of each variable and the effect of their interaction on TP removal rate were investigated by constructing response surface plots and their corresponding contour plots (Figure 3). With the increasing of dehydrated sludge dosage, the TP removal rate of PTUC showed an increasing trend. The response surface showed a steep slope (Figure 3A), which indicated the interaction between factors. The increasing of dehydrated sludge content increased the active adsorption sites for phosphate combination, which improved the TP removal rate reasonably. When the dehydrated sludge content was fixed as 4 g, the TP removal rate of PTUC showed non-significant change with the increment of cement dosage, which was about 80% (Figure 3B). It was reported that the phosphate anion will react with Ca^2+^ and OH^−^ which formed complex precipitates (Qiu et al., 2015). The addition of cement increased the Ca amount in PTNC; however, the increasing of cement dosage would decrease the porosity which hindered mass diffusion, which can be further proved by the high interaction caused by the pore forming substance sodium bicarbonate (Figures 3A,C).

In order to deter the optimum preparation conditions, the primary target value was obtained by calculating the model, and the raw materials dosage was rounded and fitted again with the model for optimal target value. PTNC was prepared consequently, and TP removal rate was tested to calculate the error and determine the fitting result (Table 5). The errors between the measured values of TP removal rate and the predicted values of the model fitting were 1.80%, indicating that the fitting effect was favorable and the model was reliable. Thus the optimal raw material ratio (per 100 g) was 84.5 g (pyrite tailings), 10 g (cement), 1 g (calcined lime), 1 g (Anhydrous gypsum), 3 g (dehydrated sludge), and 0.5 g (sodium bicarbonate).

**TABLE 5:** The optimization results of regression.

	X_1_	X_2_	X_1_	TP removal rate (%)	Degree of fitting
Primary	3.1	0.5	10	89.41	96.3
Optimal	3	0.5	10	88.55	95.5
Actual	3	0.5	10	86.96	—
Error	—	—	—	1.80%	—

### 3.4 Characterization of PTNC Prepared Under Optimized Condition

**FIGURE 4:**
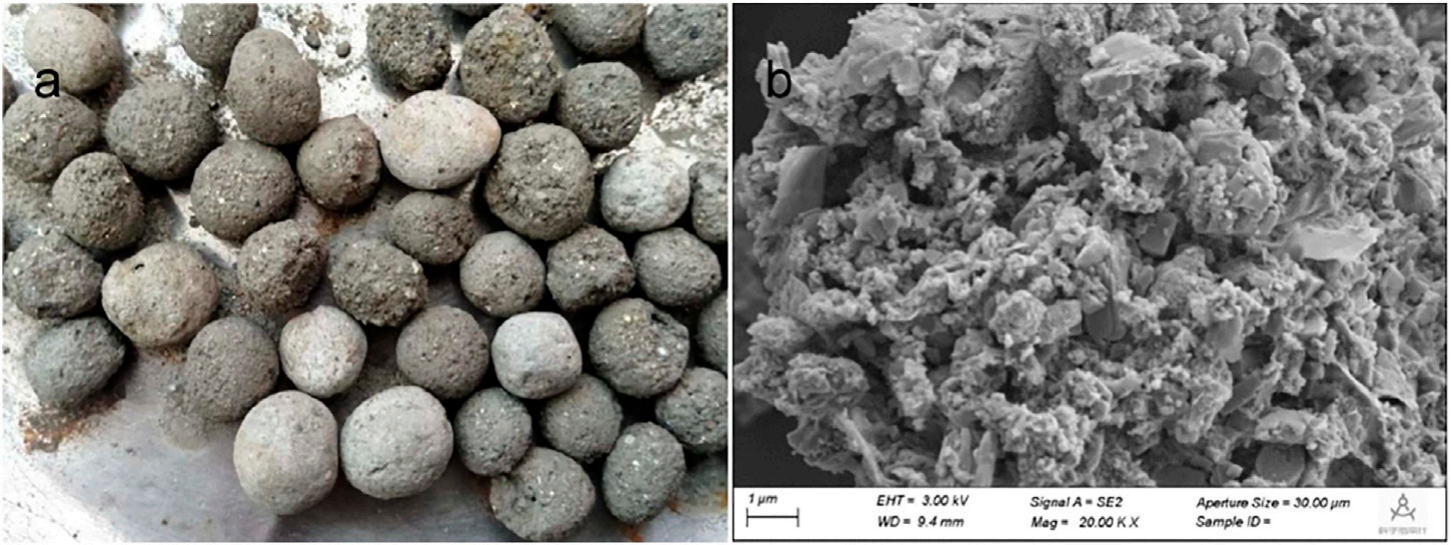
The appearance of PTNC (6–8 mm diameter) **(A)** and SEM images of PTNC **(B)**.

The surface of PTUC is rough, with low smoothness, dark gray color, and pore structure on the outer surface (Figure 4A), and the apparent morphology observed by SEM showed many grooves on its surface, with uneven interior and abundant pores, indicating its large specific surface area. The determined specific surface area and pore size was consistent with the above conclusion (Supplementary Table S5). It can be said that PTNC was suitable for the attachment and growth of microorganisms. The physical parameters of PTNC meet the requirements of relevant standards favorably (Supplementary Table S6). The high apparent density and water absorption rate ensures the adsorption characteristics of PTNC. And the favorable mechanic strength such as low wear rate and higher cylinder compressive strength ensured the application possibility of PTNC, and the porous nature is a benefit for the attachment of functional microbial community. XRD analysis confirmed the PTNC is mainly composed of FeS_2_, CaSO_4_, and SiO_2_ (Supplementary Figure S4). The peak strength of CaSO_4_ crystal is the most obvious, followed by SiO_2_, indicating the formation of SiO_2_ during the steam curing process of PTNC, acting as the framework structure of PTNC, which was also consistent with the low wear rate observed. Moreover, the heavy metal ion concentration in the PTNC leachate is much lower than the limitation of Hazardous Waste Identification Standard Leaching Toxicity Identification (GB5085.3–2007) (Supplementary Table S7), which indicate its security to the environment.

### 3.5 The Adsorption Behavior and Mechanism of TP on PTNC

**FIGURE 5:**
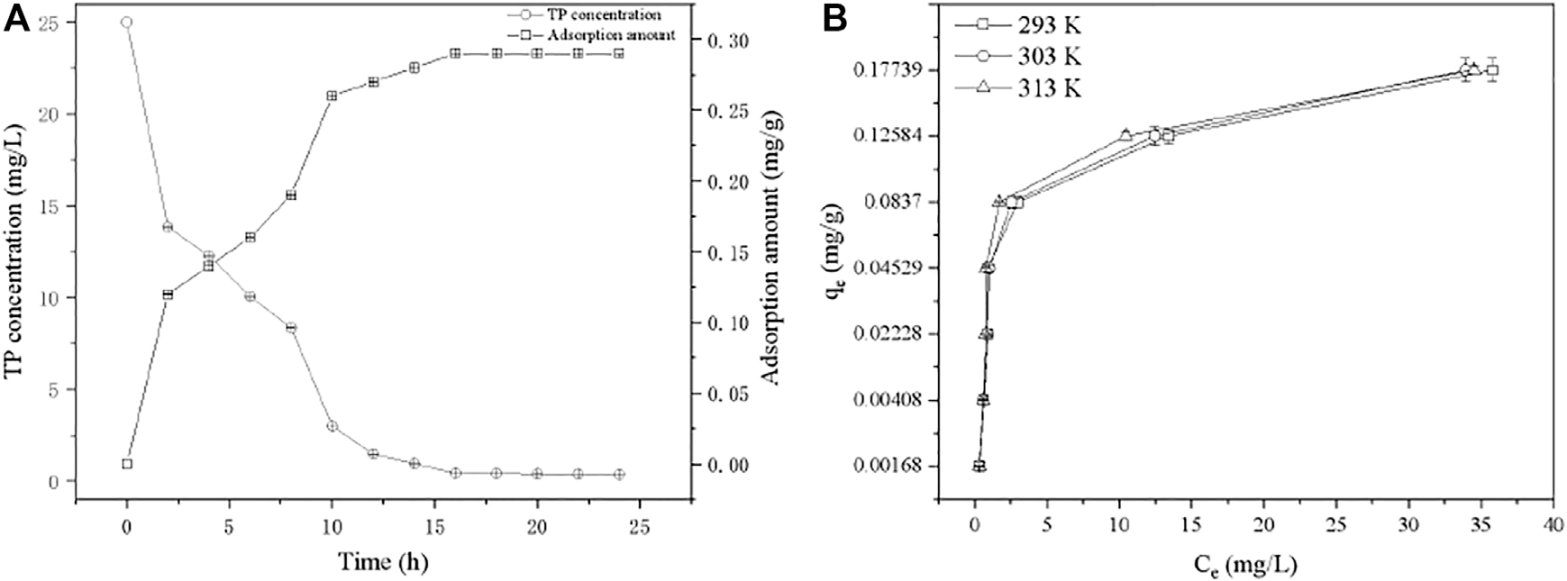
Kinetic curve **(A)** and isotherms **(B)** of TP adsorption by PTNC.

Simulated wastewater containing 25 mg/L TP was employed to investigate the adsorption kinetic. The adsorption amount showed increased tendency with the increasing of reaction time (Figure 5A). The adsorption equilibrium was observed at 16 h, where the TP concentration decreased to 0.43 mg/L, meeting the V level of “Surface water Environmental Quality Standard” (GB 3838-2002), and the equilibrium adsorption capacity was 0.2864 mg/g. As the mostly using models for description of adsorption kinetic, pseudo first-order and pseudo second-order models (Text S1) were used to fit the experimental data (Supplementary Table S8) (Chen et al., 2019). Although the correlation coefficients of both two kinetic models were similar (>0.9), the predicted adsorption capacity with pseudo first order model is 0.2995 mg/g which is close to the experimental value (0.2864 mg/g). Therefore, the pseudo first-order kinetic model can describe the TP adsorption process on PTNC better. The both two well fitted models indicated that both of chemisorption and physisorptions existed in the process of TP removal by PTNC, which were mainly ion exchange effect and precipitation probably (Yang et al., 2017). Generally, adsorption process can be divided into several steps, firstly the adsorbate in aqueous diffuses from aqueous to the adsorbent surface, then the surface loaded adsorbate diffuses to internal pores (diffusion within particles), and finally the internal adsorption sites are saturated. It can be found that the adsorption rate decreased apparently from 4 to 10 h, which can be attributed to the chemisorption of TP by PTNC leading to the formation of cobalt phosphide sulfide (Supplementary Figure S4) and decreased of the pore size of PTNC (Supplementary Table S5), thus the diffusion of phosphorus to the internal pores can be hindered.

The adsorption thermodynamics was investigated through three isotherms at different temperatures (Figure 5B), through the data fitting with Langmuir and Freundlich isotherm models (Text S2) (Chen et al., 2019). It can be found that Langmuir model fitted better with the isotherms, and indicated the mono-layer adsorption process (Supplementary Table S9). The thermodynamic parameters were calculated (Text S3), and the negative ∆G suggested the TP removal is a spontaneous process (Supplementary Table S10). Additionally, the obtained negative value of ΔH (-11.69 kJ/mol) and positive value of ΔS (153.09 J/mol/K) together indicate that the adsorption is an exothermic reaction and is both enthalpy and entropy driven. Generally, the adsorption process is an entropy reduction process; however, there are existing adsorption cases that presented entropy-positive with a negative enthalpy change (Wen et al., 2010). For TP removal process by PTNC, it can be ascribed to the releasing of Fe^3+^, Ca^2+^, and OH^−^ from PTNC surface which increased the entropy. And these ions would react with phosphate and formed of precipitation on the surface of PTNC which additionally make contribution to the entropy increasing. It can be found that the specific surface of PTNC increased obviously after adsorption (Supplementary Table S5). Moreover, the XRD analysis of the PTNC after adsorption (Supplementary Figure S4), which suggested the newly formation of cobalt phosphide sulfide, and the SEM morphology observation (Supplementary Figure S5) of the PTNC after adsorption further confirmed the precipitation formation during the adsorption process. It is consistent with the previous report (Ge et al., 2019).

**FIGURE 6:**
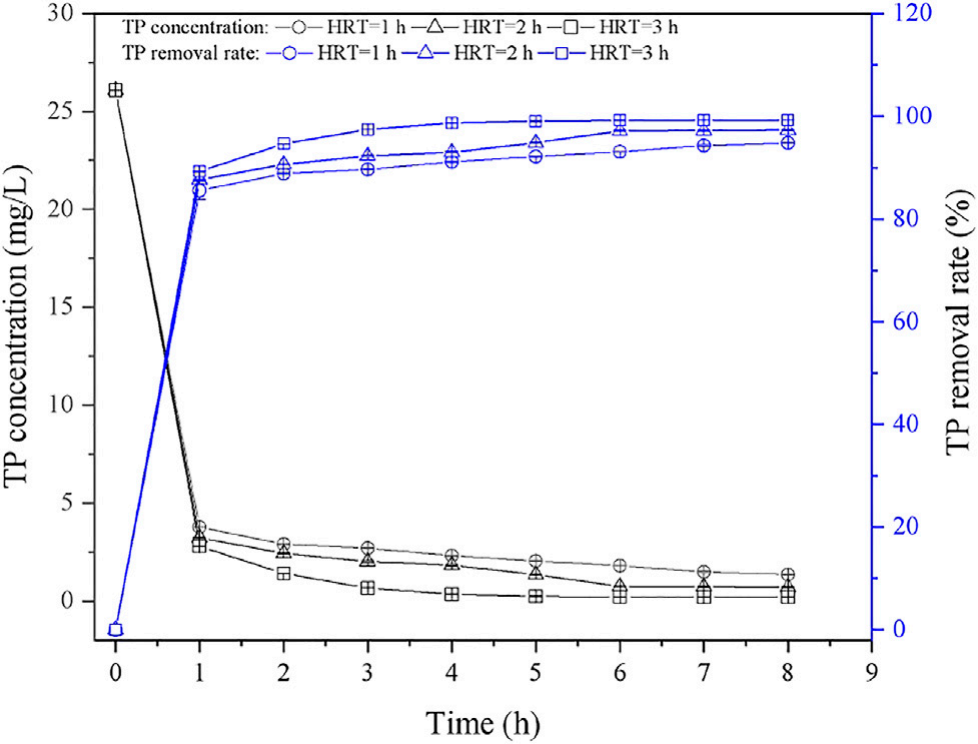
Dynamic adsorption curves of TP removal by PTNC under different HRT.

Figure 6 showed the dynamic TP removal curves under different HRT (1, 2, 3 h). In the initial stage, the TP concentration in effluent decreased rapidly, which is ascribed to the large amount of active adsorption sites in the initial stage (Chu et al., 2014). The TP concentration tended to be stable after running for 8 h, and it was due to the concentration gradient of phosphorus solution decreased gradually. In terms of HRT = 1 h, the TP concentration in the effluent was about 1.35 mg/L, and the TP removal rate was 94.82% after 8 h. As the HRT increased to 2 h, the final TP concentration in the effluent decreased to 0.69 mg/L, and the removal rate further increased to 97.34%. Similar phenomena can be observed when HRT was set as 3 h, where the stable TP concentration in the effluent is about 0.20 mg/L, which is meeting the standard of Ⅲ class surface water. As expected, the low HRT will lead to the insufficient time for phosphate contact and react with adsorption sites, as well as the released active ions; thus, suitable HRT should be considered in the application. Additionally, it can be found that the performance of PTNC was better than the phosphorus removal effect of the mixture of iron scrap and sand as previous report (88%) (Erickson et al., 2012). Table 6 showed the comparison of the TP removal ability of PTNC with other ceramsite. It can be found that PTNC presented highest TP adsorption capacity besides the Slag ceramsite reported by Liu et al. (2021). But the non-sintered preparation of PTNC is a more economical way. Thus, the prepared PTNC is promising for TP control and waste recycling.

**TABLE 6:** Comparison of the TP removal ability of different ceramsites.

Ceramsite	Calcining temperature (°C)	Initial TP concentration (mg/L	Adsorption capacity (mg/g)	Reference
CFA/WS/OS-op	1,050	100	4.51 (calculated)	Cheng et al. (2018)
Slag ceramsite	1,000	10	10.5	Liu et al. (2021)
N&P-adsorbed ceramsite	800	100	0.93	Shao et al. (2022)
DWTS ceramsite	1,050	20	1.43	Chen et al. (2019b)
PTNC	Non	25	6.9978	This work

## 4 Conclusion

In this research, a novel non-sintered ceramsite (PTNC) was prepared with pyrite tailings, and the preparation process was optimized by combination of PBD and BBD based response surface methodology, which confirmed the optimized mass ratios of the ingredients was 84.5 g (pyrite tailings), 10 g (cement), 1 g (calcined lime), 1 g (Anhydrous gypsum), 3 g (dehydrated sludge), and 0.5 g (sodium bicarbonate). PTNC presented favorable properties such as high specific surface, low leaching toxicity, and excellent mechanical strength. And the investigation on the adsorption behavior through batch and dynamic experiments showed the favorable adsorption capacity of PTNC, whose calculated q_max_ was about 7 mg/g, and can offer stable removal ability continuously under different HRT. The analysis of the adsorption mechanism suggested the existing of both physical and chemical adsorption effect. The total results make contribution to the resource of pyrite tailings, which are also benefit to the development of novel effective and economic medium for BAFS in the advanced treatment of wastewater.

## Data Availability

The original contributions presented in the study are included in the article/Supplementary Material, further inquiries can be directed to the corresponding authors.
