# Supplementary material for: Optimize the Preparation of Novel Pyrite Tailings Based Non-sintered Ceramsite by Plackett-Burman Design Combined With Response Surface Method for Phosphorus Removal
Source: Front Chem. 2022 Mar 8;10:850171. doi: 10.3389/fchem.2022.850171 (PMC8957833; doi:10.3389/fchem.2022.850171)
Supplement: Supplementary file 1 [file DataSheet1.docx]

**Optimize the Preparation of Novel Pyrite Tailings Based Non-Sintered Ceramsite (PTNC) by Plackett-Burman Design Combined with Response Surface Method For Phosphorus Removal**

Ruihuan Chen^1,2^, Zhenlin Pan^1^, Shuyi Chu^3^, Jibo Xiao^1*^, Rengui Weng^4^, Da Ouyang^5^, Yunlong Yang^1^, Xiangting Wu^1^ and Zhida Huang^6*^

^1^College of Life and Environmental Science, Wenzhou University, Wenzhou, China

^2^Zhejiang Provincial Key Laboratory for Water Environment and Marine Biological Resources Protection, Wenzhou University, Wenzhou, China

^3^Wenzhou Academy of Agricultural Sciences, Wenzhou, China

^4^Indoor Environment Engineering Research Center of Fujian Province, Fujian University of Technology, Fuzhou, China

^5^Key Laboratory of Soil Contamination Bioremediation of Zhejiang Province, School of Environmental and Resource Sciences, Zhejiang Agriculture and Forestry University, Hangzhou, China

^6^Wenzhou Institute of Industry and Science, Wenzhou, China

*Co-corresponding authors:

Jibo Xiao [(jbxiao@wzu.edu.cn);](mailto:(jbxiao@wzu.edu.cn);)

Zhida Huang [(40098008@qq.com)](mailto:(40098008@qq.com))

**Acknowledgments:** The authors appreciate all the participants.

**Text S1** Adsorption kinetic models

Nonlinear form pseudo fist-order:

$\text{q}_{\text{t}}\text{=}\text{q}_{\text{e}}\text{(1-}\text{e}^{\text{-kt}}\text{)}$ (S1)

where q_t_ is the adsorption amount at time t (mg/g), q_e_ is the equilibrium adsorption amount (mg/g), and k_1_ is the equilibrium rate constant of the model (min^-1^).

Nonlinear formula of pseudo second-order model:

$\text{q}_{\text{t}}\text{=}\frac{\text{q}_{\text{e}}^{\text{2}}\text{k}_{\text{2}}\text{t}}{\text{1+}\text{q}_{\text{e}}\text{k}_{\text{2}}\text{t}}$ (S2)

where k_2_ is the pseudo second-order rate constant (mg/g/min).

**Text S2** Adsorption isotherm models

Langmuir empirical model:

$\text{q}_{\text{e}}\text{=}\frac{\text{q}_{\text{m}}\text{k}_{\text{L}}\text{C}_{\text{e}}}{\text{1+}\text{k}_{\text{L}}\text{C}_{\text{e}}}$ (S3)

where C_e_ (mg/L) is the equilibrium adsorbate concentration in liquid, q_m_ and k_L_ are the Langmuir maximum adsorption capacity and the energy related constant respectively.

The Freundlich isotherm is described as the following equation:

$\text{q}_{\text{e}}\text{=}\text{k}_{\text{F}}\text{C}_{\text{e}}^{\text{1}/\text{n}}$ (S4)

where k_F_ is the Freundlich constant and n is the heterogeneity factor (Freundlich coefficient).

**Text S3** Thermodynamic parameters calculation method

The free energy of adsorption (∆G), enthalpy of adsorption (∆H), and entropy of adsorption (∆S) during the adsorption process by PTNC was be calculated as with following equations:

$\text{∆G=-RT}\ln\text{K}$ (S5)

$\ln\text{K}\text{=}\frac{\text{∆S}}{\text{R}}\text{-}\frac{\text{∆H}}{\text{RT}}$ (S6)

Where R is the universal gas constant, 8.314 (J/mol/K); T (K) is the absolute solution temperature; K is the dimensionless thermodynamic equilibrium constant. The ΔH and ΔS values are calculated from the slope and intercept of the plot with the plot of lnK versus 1/T. Before the calculation process, the K gained from the best fitted model (Langmuir isotherm) need to be recalculated by multiplying it by 10^6^ to become dimensionless, due to its unit was L/mg.

**Table S1** Chemical composition of original pyrite tailings

| Composition | S | Fe | Si | P | C | Mn |
| --- | --- | --- | --- | --- | --- | --- |
| Percent (%) | 45.32 | 50 | 2.2 | 0.23 | 0.26 | 0.4 |

**Table S2** Chemical composition of dehydrated sludge

| Composition | SiO_2_ | Al_2_O_3_ | Fe_2_O_3_ | CaO | MgO | Na_2_O | K_2_O |
| --- | --- | --- | --- | --- | --- | --- | --- |
| Percent (%) | 63.51 | 17.21 | 6.53 | 0.96 | 3.65 | 1.71 | 2.89 |

**Table S3** List of levels and factors of Plackett-Burman experiment

| Factors | Code | Levels | |
| --- | --- | --- | --- |
|  |  | -1 | +1 |
| Cement | A | 10 | 30 |
| Calcium lime | B | 1 | 3 |
| Anhydrous gypsum | C | 1 | 3 |
| Dehydrated sludge | D | 2 | 6 |
| Sodium bicarbonate | E | 0.5 | 1.5 |

**Table S4** Variables and levels in Box-Behnken design response surface method

| Levels | Factors | | |
| --- | --- | --- | --- |
|  | X_1_  Dehydrated sludge (g) | X_2_  Sodium bicarbonate (g) | X_3_  Cement (g) |
| -1 | 2 | 0.5 | 10 |
| 0 | 4 | 1 | 20 |
| +1 | 6 | 1.5 | 30 |

**Table S5** Analysis results of specific surface area and pore size with BET method

| Parameters | Units | Original PTNC | PTNC after adsorption |
| --- | --- | --- | --- |
| Specific surface area | m²·g^-1^ | 7.21 | 11.39 |
| Average pore volume | cm³·g^-1^ | 0.024 | 0.029 |
| Average pore aperture | nm | 13.42 | 10.08 |

**Table S6** Physical characteristics of PTNC

| Indicators | Standard value | PTNC performance |
| --- | --- | --- |
| Water adsorption (%) | 22 | 25.08 |
| Cylinder compressive strength (MPa) | 1.0 | 1.80 |
| Porosity (%) | ≥40 | 53.90 |
| Solubility in hydrochloric acid (%) | ≤2 | 5.36 |
| Specific surface area (m^2^/g) | 0.5 | 7.21 |
| Wear rate (%) | ≤6 | 2.08 |
| Silt content (%) | ≤1 | 0.54 |

**Table S7** The concentrations of heavy metals in the PTNC leachate

|  | Cu | Pb | Cr | Zn | Ni | Cd |
| --- | --- | --- | --- | --- | --- | --- |
| Detected value (mg/L) | 0.096 | 0.003 | 0.007 | 0.258 | 0.002 | 0.012 |
| Standard limitation （mg/L） | 100 | 5 | 5 | 100 | 5 | 1 |

**Table S8** The fitted parameters of two adsorption dynamic models

| Pseudo first order | | | Pseudo second order | | |
| --- | --- | --- | --- | --- | --- |
| q_e1_ (mg/g) | k_1_ (1/h) | R^2^ | q_e2_ (mg/g) | k_2_ (g/mg/h) | R^2^ |
| 0.2995 | 0.1661 | 0.9155 | 0.3527 | 0.4691 | 0.9100 |

**Table S9** The isothermal adsorption model parameters of TP adsorption

| T (K) | Langmuir | | | Freundlich | | |
| --- | --- | --- | --- | --- | --- | --- |
|  | q_m_ (mg/g) | K_L_ (L/mg) | R^2^ | K_F_ (L/mg) | n (L/g) | R^2^ |
| 293 | 6.9603 | 0.8543 | 0.9421 | 3.0483 | 0.2487 | 0.8348 |
| 303 | 6.9824 | 0.8678 | 0.9574 | 3.0880 | 0.2495 | 0.8382 |
| 313 | 6.9978 | 1.1648 | 0.8530 | 3.2436 | 0.2353 | 0.7875 |

**Table S10** Thermodynamic parameters for TP adsorption by PTNC

| T (K) | ∆G (kJ/mol) | ∆H (kJ/mol) | ∆S (J/mol/K) |
| --- | --- | --- | --- |
| 293 | -33.27 | -11.69 | 153.09 |
| 303 | -34.45 |  |  |
| 313 | -36.35 |  |  |

**Fig. S1** XRD pattern of pyrite tailings


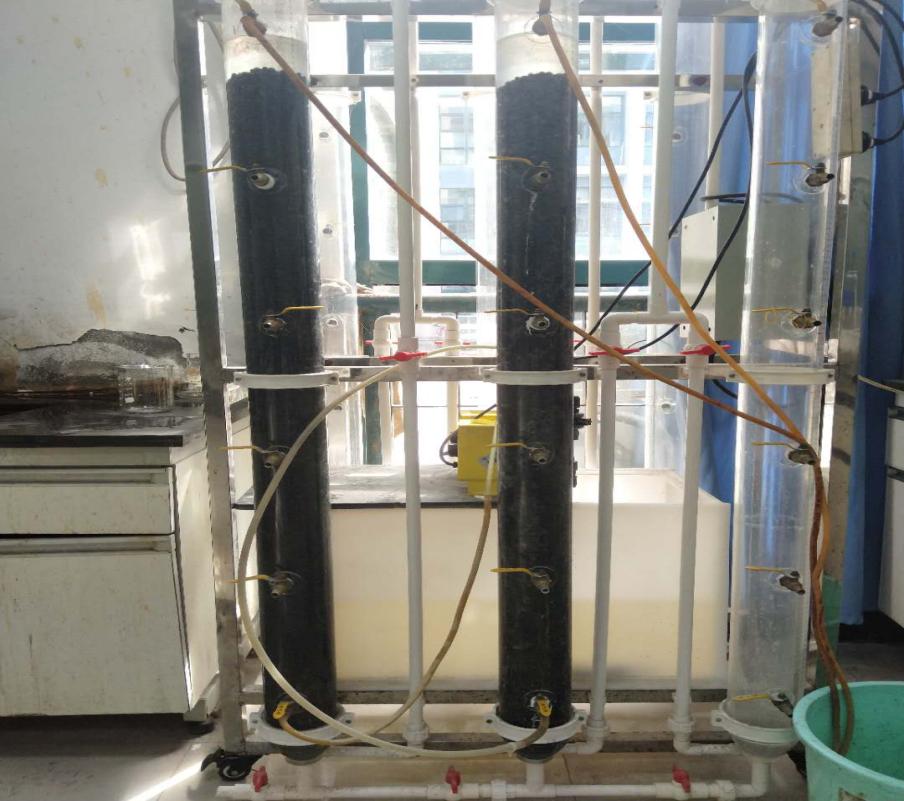


**Fig. S2** Experimental device for dynamic adsorption experiment

**Fig.S3** Comparison analysis of experimental and predicted TP removal rates

**Fig. S4** Comparison XRD analysis of pyrite tailings, PTNC before and after adsorption of TP.


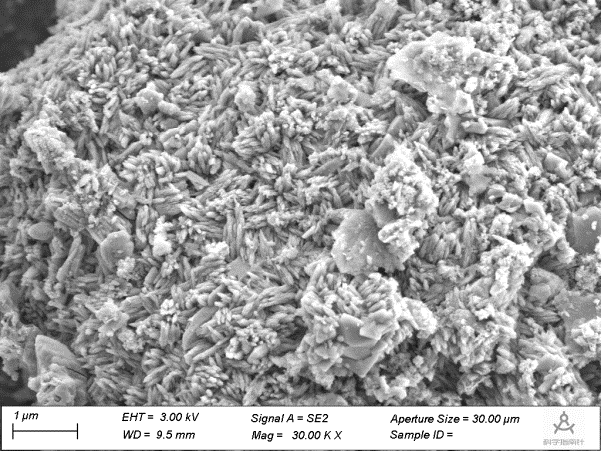


**Fig. S5** SEM image of PTNC after adsorption
